# Infrared absorbance spectroscopy of aqueous proteins: Comparison of transmission and ATR data collection and analysis for secondary structure fitting

**DOI:** 10.1002/chir.23002

**Published:** 2018-07-20

**Authors:** Marco Pinto Corujo, Meropi Sklepari, Dale L. Ang, Mark Millichip, Andrew Reason, Sophia C. Goodchild, Paul Wormell, Don Praveen Amarasinghe, Viv Lindo, Nikola P. Chmel, Alison Rodger

**Affiliations:** ^1^ Department of Chemistry University of Warwick Coventry UK; ^2^ MOAC and MAS Centres for Doctoral Training University of Warwick Coventry UK; ^3^ School of Science and Health Western Sydney University Penrith New South Wales Australia; ^4^ BioPharmaSpec Ltd. Jersey UK; ^5^ Department of Molecular Sciences Macquarie University North Ryde New South Wales Australia; ^6^ MedImmune Ltd Cambridge UK

**Keywords:** attenuated total reflectance, ATR correction, refractive index, depth of penetration

## Abstract

Attenuated total reflectance (ATR) infrared absorbance spectroscopy of proteins in aqueous solution is much easier to perform than transmission spectroscopy, where short path‐length cells need to be assembled reproducibly. However, the shape of the resulting ATR infrared spectrum varies with the refractive index of the sample and the instrument configuration. Refractive index in turn depends on the absorbance of the sample. In this work, it is shown that a room temperature triglycine sulfate detector and a ZnSe ATR unit can be used to collect reproducible spectra of proteins. A simple method for transforming the protein ATR spectrum into the shape of the transmission spectrum is also given, which proceeds by approximating a Kramers‐Krönig–determined refractive index of water as a sum of four linear components across the amide I and II regions. The light intensity at the crystal surface (with 45° incidence) and its rate of decay away from the surface is determined as a function of the wave number–dependent refractive index as well as the decay of the evanescent wave from the surface. The result is a single correction factor at each wave number. The spectra were normalized to a maximum of 1 between 1600 cm^−1^ and 1700 cm^−1^ and a self‐organizing map secondary structure fitting algorithm, SOMSpec, applied using the BioTools reference set. The resulting secondary structure estimates are encouraging for the future of ATR spectroscopy for biopharmaceutical characterization and quality control applications.

## 1 INTRODUCTION

Spectroscopic techniques measure the interaction of radiation with matter and are loosely separated from microscopy techniques in that they usually involve scanning over a wavelength/frequency/energy range and typically average over many molecules at one time with little spatial resolution. The fact that spectroscopic measurements average over all species through which the light beam passes—a 1 mM sample in a 1 cm path length cell with a 1‐mm^2^ light beam contains 10^15^ molecules—means that we may need to change variables such as concentration or temperature or solvent or sample preparation more generally to determine spectra for single species, but it is usually possible.

A current challenge is to determine whether different biopharmaceutical formulations of a drug are the same. This has come into focus as the patents of an increasing number of biopharmaceuticals are expiring, creating the opportunity to develop so‐called biosimilar drugs.^1^ We desperately need analytical methodologies to determine how “similar” a proposed product is to the original innovator product. In contrast to small molecule drugs, the activity of a protein biopharmaceutical is dependent not only on its primary structure (what atom is bonded to what) but also on its secondary and tertiary structures. Within any solution‐phase sample, there will be a distribution of geometries, either in equilibrium and so on average the same or in actually different isolatable structures, so it is important to work with the actual product rather than a purified version of it.

Circular dichroism (CD) spectroscopy has been gradually accepted as a means of estimating the secondary structure of unknown proteins in the biopharmaceutical arena. There are a range of methods to extract the secondary structure content of a protein sample from its CD spectrum by comparing with a reference set of spectra of known secondary structures.^2^, ^3^, ^4^ However, any absorption spectroscopy technique has a dynamic range limited by the need to have enough photons reaching the detector for us to be able to count. In practice, for protein CD, this means the protein plus anything else in the solution should have an absorbance between about 0.3 and 2.5, and preferably about 1. Given the Beer‐Lambert law

(1) A=εCl

for *A* being absorbance, *ε* the wavelength dependent extinction coefficient (which depends on all the electronic structural information), *C* the concentration, and *l* the path length. Even in water, we are limited to a maximum of *Cl* = 0.02 mg/cm^2^ (equivalent to 0.2 mg/mL protein in a 1 mm cuvette) and arguably less. The excipients required to hold the proteins in solutions at high concentration typically include chiral amino acids and sugars making the cell assembly extremely important as the buffers have a significant CD spectrum. Thus, CD is not the ideal technique for highly concentrated samples or samples formulated with other highly absorbing molecules.

An alternative strategy is to consider the infrared (IR) region of the spectrum: while allowed electronic transitions typically have extinction coefficients of up to 20,000 mol^−1^ cm^−1^ dm^3^, IR samples are seldom greater than 100 mol^−1^ cm^−1^ dm^3^. Infrared spectra give the energies and intensities of the molecule's vibrational modes, which for proteins do contain information about secondary structure.^5^, ^6^ We therefore sought to complement CD with Fourier‐transform IR spectroscopy, but were surprised to find that our expectation of readily finding robust‐validated protocols for data collection and protein structure fitting was naive.^7^ Our early work used transmission spectroscopy, but we found it very difficult to collect reproducible aqueous protein spectra and appropriate buffer baseline spectra for reasons discussed below.

We then moved to using attenuated total reflectance (ATR)‐IR spectroscopy in H_2_O, where our data collection was much improved, but a spectrum collected using an ATR‐IR unit has wavelength and refractive index dependence,^8^, ^9^, ^10^ which translates into wavelength and absorbance dependence, so the spectrum is a function of the instrument, the sample, its concentration, and buffer components (even when they do not affect the protein structure). The goal of this work was therefore to develop methods for using ATR‐IR in protein structure fitting. It involved both experimental protocols and data analysis methods to convert ATR‐IR spectra to the spectral shape of the corresponding transmission spectrum, which is instrument independent (assuming the instrument is calibrated) and follows the Beer‐Lambert law.

## 2 MATERIALS AND METHODS

### 2.1 Protein samples

Standard protein solutions of hemoglobin (L7647, Sigma), concanavalin A (H2500, Sigma), and lysozyme (62970, Sigma Aldrich) were prepared by dissolving lyophilized powder in water (18.2 MΩ) at the concentrations stated.

### 2.2 Transmission IR data collection

Transmission IR spectra of hemoglobin (approximately 100 mg/mL), concanavalin A (approximately 20 mg/mL), lysozyme (approximately 100 mg/mL), and ultrapure water were collected using a Jasco FVS‐6000 vibrational CD spectrometer and a Jasco FT/IR‐4700 spectrometer with a mercury cadmium telluride detector and PIKE Technologies demountable liquid cell. Samples (6 μL) were placed on one of the CaF_2_ cell windows before the cell was assembled with a 4 μm spacer. Five hundred scans, from 850 to 2000 cm^−1^ with a resolution of 4 cm^−1^, were collected. Repeat spectra were collected until protein and water spectra that overlaid in the 2125 cm^−1^ region were obtained, which also had similar levels of water vapor.

### 2.3 ATR‐IR protein data collection

Attenuated total reflectance IR spectra of hemoglobin (10 mg/mL), concanavalin A (10 mg/mL), lysozyme (10 mg/mL), and water were collected using a Jasco FT/IR‐4700 with triglycine sulfate (TGS) detector and PIKE Technologies MIRacle single reflection ATR accessory with the spectrometer sample compartment left open. A background scan was performed immediately prior to each sample or water measurement. Five microliters of sample were placed directly on the ZnSe crystal plate, and 64 scans, 3000 to 800 cm^−1^ with a resolution of 4 cm^−1^, were collected. The difference between repeat spectra of the clean, dry ATR crystal was collected to give a water vapor signal to be used for water vapor correction. The crystal was cleaned with water and a lens tissue, and we ensured there was no remaining protein signal by measuring an “air” spectrum.

## 3 RESULTS AND DISCUSSION

In this work, we selected stable, robust, proteins whose structure does not change with concentration and which represents highly α‐helical (hemoglobin), highly β‐sheet (concanavalin), and moderately helical structures (lysozyme) to explore our ability first to collect data and second to determine secondary structures.

### 3.1 Transmission protein data collection

**Figure 1:**
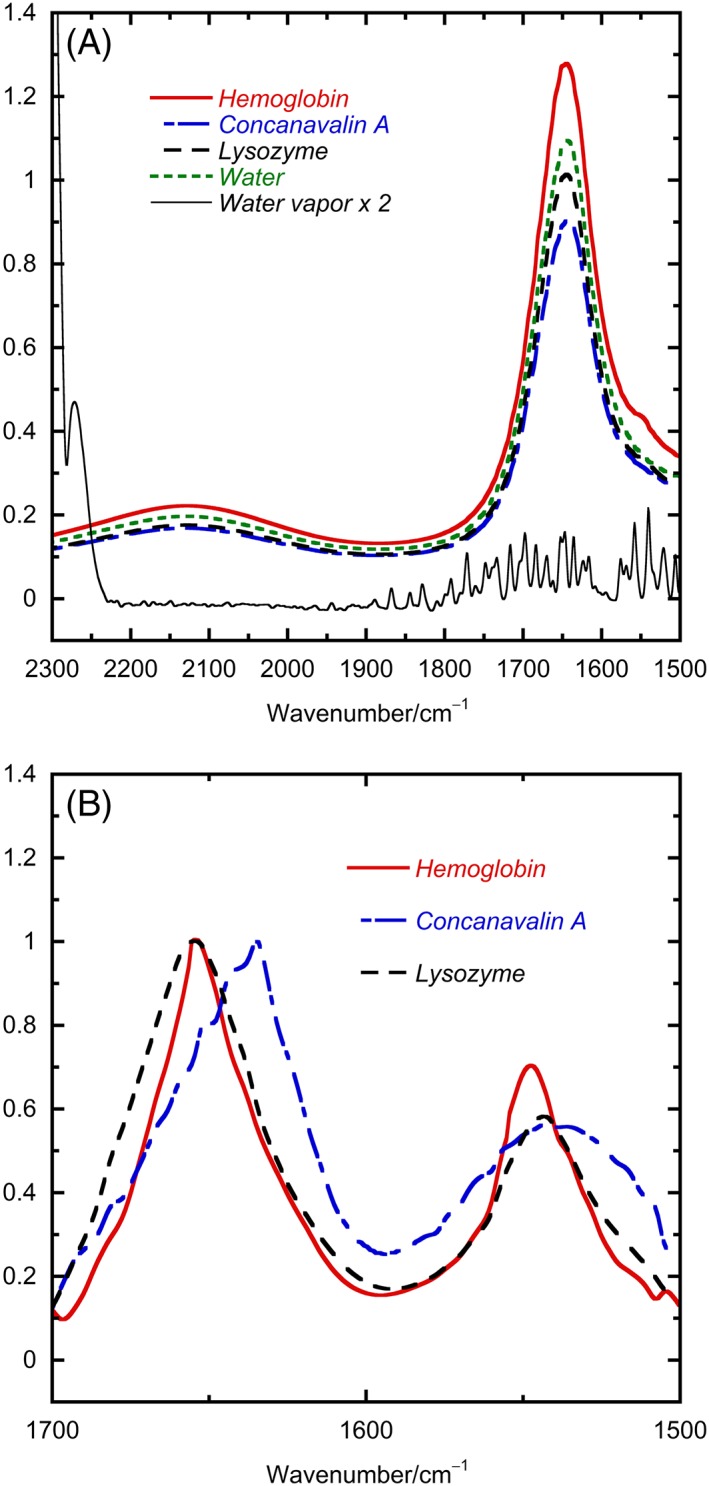
A, Transmission absorbance spectra of water (55M), hemoglobin (approximately 100 mg/mL), concanavalin (approximately 20 mg/mL), and lysozyme (approximately 100 mg/mL) in transmission mode with a cell with 4 μm spacer but cell assembled to 7–10 μm path length. B, Water baseline–corrected spectra from (A) normalized to a maximum of 1

The first part of our work was to collect transmission spectra, on the same samples as for our proposed ATR experiments, that could be used as standards to indicate whether our protocol to transform ATR‐IR spectra into transmission spectra was working. The structures of our chosen proteins do not change with concentration, and protein IR data are normalized to 1 before structure fitting, so approximate concentrations were sufficient. Figure 1A shows transmission IR data for water (55 M and *ε* ~ 21.7 mol^−1^ cm^−1^ dm^3^ at 1643 cm^−1^)^11^ and aqueous solutions of proteins representing highly α‐helical, moderately α‐helical, and β‐strand structures at high concentration with a nitrogen‐purged interferometer and demountable CaF_2_ cells assembled using a 4 μm Teflon spacer. Here, we either used a highly nitrogen‐purged vibrational CD instrument with a mercury cadmium telluride detector so we could ignore the contribution from water vapor or performed multiple repeat experiments in an unpurged Jasco FT/IR‐4700 until we had pairs of sample and water spectra with matching water and water vapor signals at 2125 and 1700 cm^−1^, respectively, as discussed below. Figure 1A shows a set of such spectra. The spectra are all dominated by water absorbance, so the magnitude variation is mainly due to differences in path lengths—despite our best efforts, we could not assemble a 4 μm path length cell accurately, and it varied from 7.5 to 10 μm path length. The protein amide I band occurs between 1700 and 1600 cm^−1^ and the amide II band between 1600 and 1500 cm^−1^. These bands are essentially invisible under the water‐bending vibration in Figure 1A, which spans the 1500‐ to 1700‐cm^−1^ region.

The amide I band is generally deemed to reflect the secondary structure of proteins,^5^, ^6^ so this is the information we needed to extract for biopharmaceutical products formulated in aqueous media. Great care must be taken in subtracting the baseline as we are looking for a small difference between two large spectra. We used the 2125‐cm^−1^ region combination bend + libration band of liquid water^12^ to indicate when we had successfully subtracted the liquid water spectrum as there is no water vapor or protein signal in this region.^5^ We usually used the subtraction option in the Jasco software as it lets one scale the water spectrum in steps of 0.001. If it was not possible to render the spectrum flat between 2000 and 2300 cm^−1^ we discarded that data set. With good data sets, different operators achieve the same final buffer‐corrected spectrum. We also used as high a protein concentration as possible to increase the protein contribution relative to that of water. A set of the resulting baseline‐corrected spectra, normalized to a maximum of 1 to facilitate comparison, are shown in Figure 1B. The β‐sheet concanavalin A amide I maximum occurs at lower wave numbers than that of the helix‐containing proteins, and the higher helix content hemoglobin has a narrower envelope than the mixed structure lysozyme. The amide II band has approximately 60% of the intensity of the amide I band for the three spectra. However, it should be noted that the intensity and shape of the amide II band can be strongly influenced by the presence of cations and anions,^13^ which are often present at high concentrations in formulation vehicles.

### 3.2 ATR‐IR protein data collection

**Figure 2:**
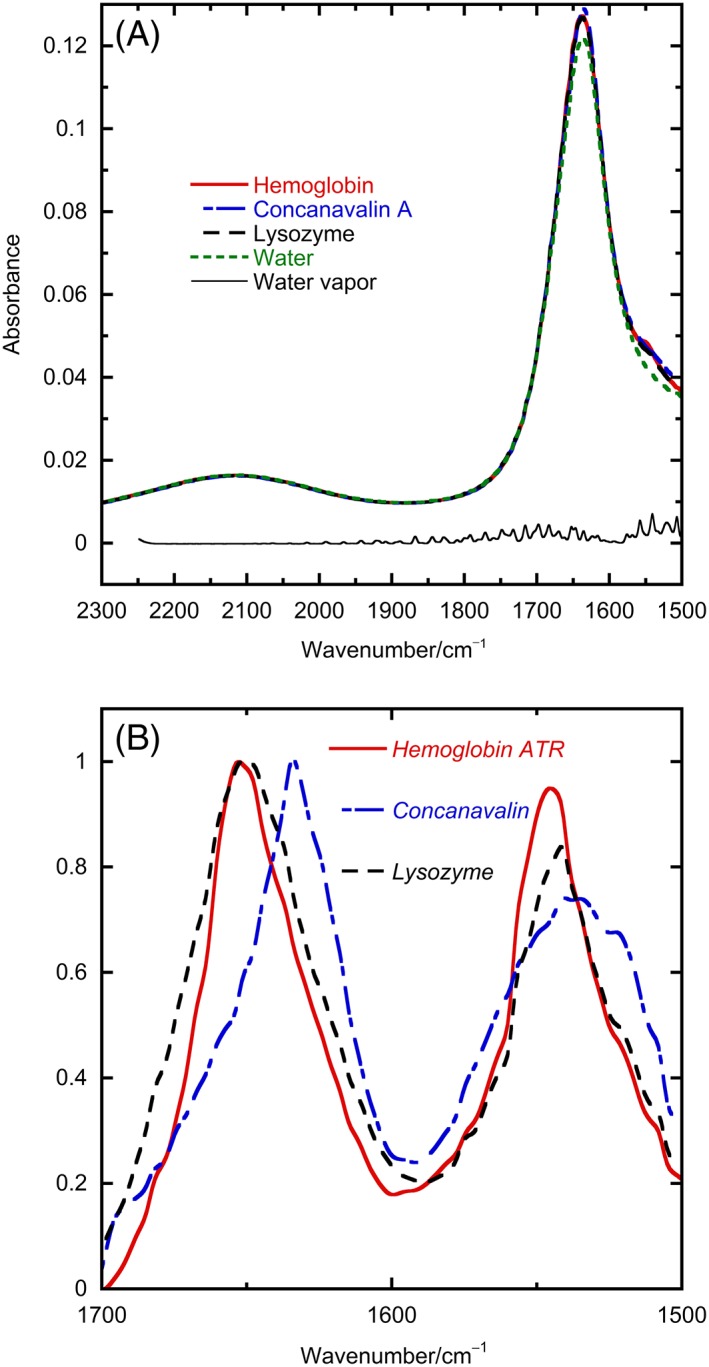
A, Attenuated total reflectance spectra of water (55M), hemoglobin (10 mg/mL), concanavalin (10 mg/mL), and lysozyme (10 mg/mL) with a ZnSe ATR unit and 45° angle of incidence. B, Baseline‐corrected protein spectra from (A) scaled to a maximum of 1

Figure 2 shows data analogous to Figure 1 (but for 10 mg/mL samples) for data collected with an ATR unit inserted into the light beam of an absorbance IR spectrometer, in this case without any nitrogen purging. These alternative means of IR absorbance data collection are widely used for small molecules. The unit inverts the light beam into a dense crystal, which totally internally reflects the beam but, because of various conservation laws as summarized in Maxwell equations,^10^ results in an electric field above the surface of the crystal—often referred to as an evanescent wave—which decays exponentially from the surface. Thus, an absorbing sample placed on the crystal may interact with the light's electric field, causing absorbance. A liquid sample for such an experiment is simply dropped onto the crystal, and data collection begun. Care does need to be taken to avoid evaporation.

In our experiments, we chose a ZnSe ATR crystal, with 45° incidence angle and a TGS detector. No ATR crystal is perfect, and we chose ZnSe because of its higher energy transmission than diamond and its deeper penetration depth than germanium that gives more intense signals and means that the effect of any surface ordering is reduced compared with Ge; 45° incidence with ZnSe runs the risk of losing total internal reflectance if the sample refractive index drops below 1.2. However, this does not occur in our experiments. The presented data are reproducible from run to run apart from the presence of more or less water vapor. We noticed no increase in signal over time as would be expected if the protein was being concentrated at the surface of the crystal, unless the water was also seen to evaporate. A reduction in protein concentration to 1 mg/mL (data not shown) is possible although replicates are not completely reproducible.

Baseline correction was undertaken by subtracting a scaled (usually with a factor between 0.97 and 1.01) water spectrum to achieve a flat region around 2125 cm^−1^ as discussed above for the transmission spectra. We then added or subtracted a scaled water vapor spectrum (itself created as the difference of two “air” spectra) until the water vapor bands around 1700 cm^−1^ disappeared. By running a background just before each spectrum in an air‐conditioned laboratory, we found that we could normally manage without nitrogen purging and with little water vapor correction.

### 3.3 Comparison of transmission and ATR data

**Figure 3:**
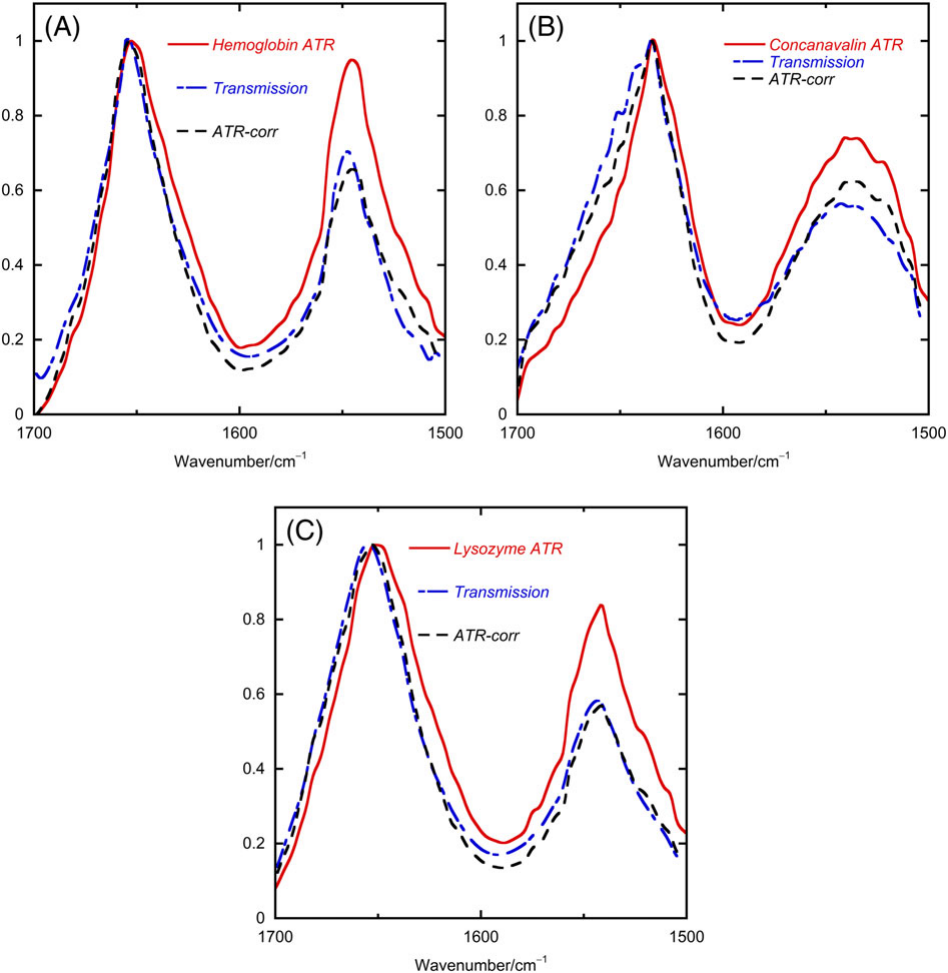
Baseline‐subtracted spectra for, A, hemoglobin, B, concanavalin A, and, C, lysozyme all normalized to a maximum of 1 in the amide I band. ATR, attenuated total reflectance

Although Figure 2A spectral shapes look similar to Figure 1A data, Figures 2B and 1B data are clearly different with slightly different band shapes and the 2B data being relatively larger for the amide II band than for the amide I. Figure 3 shows an overlay of the buffer‐subtracted protein spectral data from Figures 1 and 2 together with the results of the ATR correction process outlined below. The ATR‐corrected (ATR‐corr) spectra are all similar to the transmission spectra over the amides I and II regions, with similar meaning as close as repeat buffer‐corrected transmission spectra are to each other in our hands. By way of contrast, the ATR and transmission spectra differ significantly. The underlying reason for this difference is that the sample affects the magnitude of the electric field of the light above the surface of the ATR crystal. We can separate this influence into two aspects requiring correction:

- how the sample affects the rate of decay of the electric field from the surface, and
- the magnitude of the electric field at the surface.

The penetration depth or effective path length is a parameter that lets us think in terms of the linear Beer‐Lambert law dependence of signal on concentration, extinction coefficient, and path length by putting all the complexities of the first correction (the rate of decay of the field) into an effective path length. However, it is not a simple path length as discussed below but depends on wave number and sample absorbance. The second correction is a further complication as it also depends on sample absorbance in a different way as well as instrument configuration.

### 3.4 Implementation of corrections to transform protein ATR spectra into transmission spectra

Although equations that describe the wave number dependence of penetration depth are available in the literature, we were unable to find their derivation, which means we were unable to understand how to correct protein ATR‐IR data in a manner that we could defend for biopharmaceutical products. We therefore derived the required equations^14^ and in this work implement an approximation to them for our application. The decay of the light's electric field from the surface and the consequent direct wavelength dependence of the penetration depth are summarized in the well‐known depth of penetration.^10^

(2) $$d_{p}=\frac{1}{\alpha }=\frac{\lambda_{0}}{2\pi n_{i}\sqrt{\sin^{2}\theta_{i}-n_{t}^{2}/n_{i}^{2}}},$$

where *n* is refractive index, *i* refers to incident light, *t* refers to light transmitted above the surface, 0 refers to vacuum, *λ* is the wavelength of light (the inverse of the wave number), and *θ*_*i*_ is the angle of incidence of the light.

To correct for the wave number dependence of how far into the sample the light penetrates, we therefore divide our spectrum by Equation (2). The biggest influence on *d*_*p*_ is its proportionality to wavelength making the light penetration at 1600 cm^−1^ ~ 6% greater than that at 1700 cm^−1^. There is also a dependence on the crystal and sample refractive indices and the angle of incidence (45° in our experiments). The sample refractive index is in turn a function of the absorbance as discussed below, varying from sample to sample and wave number to wave number.

Another factor that is usually ignored, unless polarized light spectroscopy is being considered, is that the magnitude of the electric field at the crystal surface is a function of the crystal and sample refractive indices. This factor is also significant for unpolarized ATR experiments if the sample absorbance is high. Absorbance is defined as

(3) $$\alpha =\frac{\text{total energy absorbed}\;\mathrm{per}\;\text{unit time}}{\text{total incident intensity}\;\left(\text{energy}/\text{unit time}/\text{area}\right)}$$

In transmission spectroscopy, the light intensity incident on the sample is the same as on an empty sample compartment, which we account for with the baseline correction. However, in ATR spectroscopy, the nature of the sample (in particular, its refractive index) alters the intensity of the light at the surface of the crystal. For a 45° incidence ATR ZnSe unit with unpolarized light and an unoriented sample, the light intensity just above the surface of the crystal is proportional to^14^

(4) $$I_{t0}\left(z=0\right)\;\alpha \;n_{t}n_{i}^{2}\left[\frac{\left(n_{i}^{2}+n_{t}^{2}\right)}{{\left(n_{i}^{2}-n_{t}^{2}\right)}^{2}}\right]E_{i0}^{2},$$

where *E*_*i*0_ is the electric field incident from beneath onto the crystal surface. As we are scaling the data to 1 for use in structure fitting and Equation (4) is constant for air, we can correct for the magnitude of the electric field at the surface by dividing the measured ATR spectrum by

(5) $$n_{t}n_{i}^{2}\left[\frac{\left(n_{i}^{2}+n_{t}^{2}\right)}{{\left(n_{i}^{2}-n_{t}^{2}\right)}^{2}}\right]$$

for the sample of interest (thus dealing with correction (ii)).

**Figure 4:**
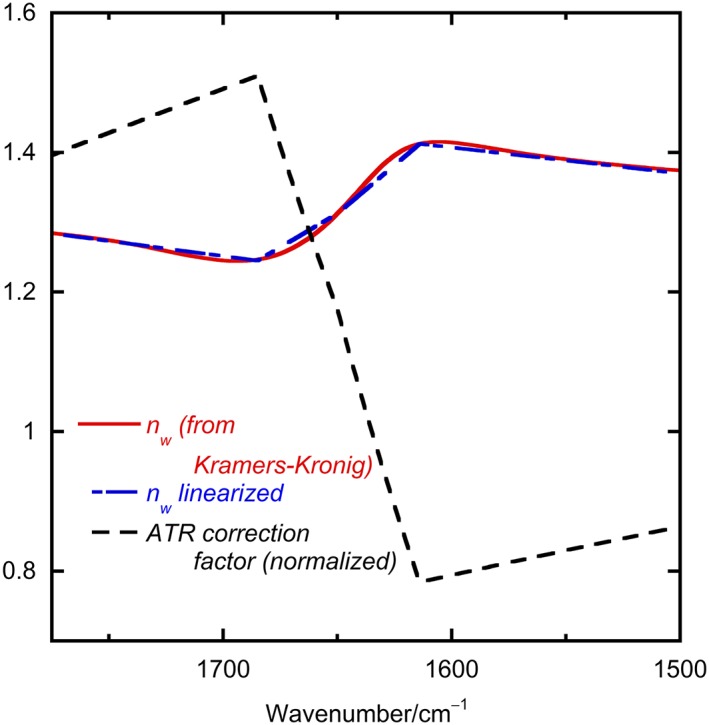
Wavelength dependence of the refractive index of water in the amides I and II regions overlaid with the correction factor used to correct aqueous protein attenuated total reflectance (ATR) spectra to resemble transmission spectra

To evaluate Equations (2) and (5), we need *n*_*t*_ as a function of wave number. For aqueous protein samples, the water and protein samples have very similar absorbances, so to a first approximation, we can ignore the difference and use the wave number dependence of the water's refractive index for both sample and baseline. Water's refractive index can be determined from its transmission absorbance using a Kramers‐Krönig transformation.^15^ *n*_*t*_ for water is illustrated^14^ in Figure 4 overlaid with a linear approximation to it (useful as IR instruments all seem to have different wave number starting points):
(6)
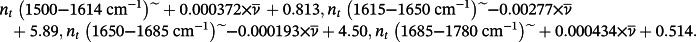



To correct the shape of the ATR spectra for the amount of light the samples “sees” (corrections (i) and (ii)), we therefore multiply the ATR experimental spectrum by the inverse of Equations (2) and (5), evaluated using Equation (6). The wave number dependence of the scaling factor is also plotted in Figure 4. The ATR‐corr spectra of Figure 3 are the result of multiplying the buffer‐corrected normalized ATR spectra with the correction factor for each wave number. An Excel spreadsheet may be found in the Supporting Information to facilitate this process.

### 3.5 Extracting protein secondary structure from an IR spectrum

Extracting secondary structure content of proteins from IR data is typically done by band fitting with the components at 1645 to 1660 cm^−1^ attributed to α‐helix, at 1620 to 1640 and 1670 to 1695 cm^−1^ to β‐sheet, at 1620 to 1640 and 1650 to 1695 cm^−1^ to turns, and at 1640 to 1657 and 1660 to 1670 cm^−1^ to other structures.^7^ Alternatively, the fitting is done on the second derivative spectrum.^6^ One then assumes the relative areas are the relative weightings of the structures. In our hands, this approach can work reasonably well if the spectrum has good signal to noise, the buffer subtraction is perfect, and the spectrum has no unusual features. However, our fits vary noticeably from attempt to attempt. The BioTools PROTA‐3S software uses a factor analysis approach to extract protein structure from IR data with their own data base of 47 spectra for proteins of known structure. In our hands, this worked for high‐quality data but not for low‐concentration samples with lower quality data. So we turned to the CD community's approaches.

**Figure 5:**
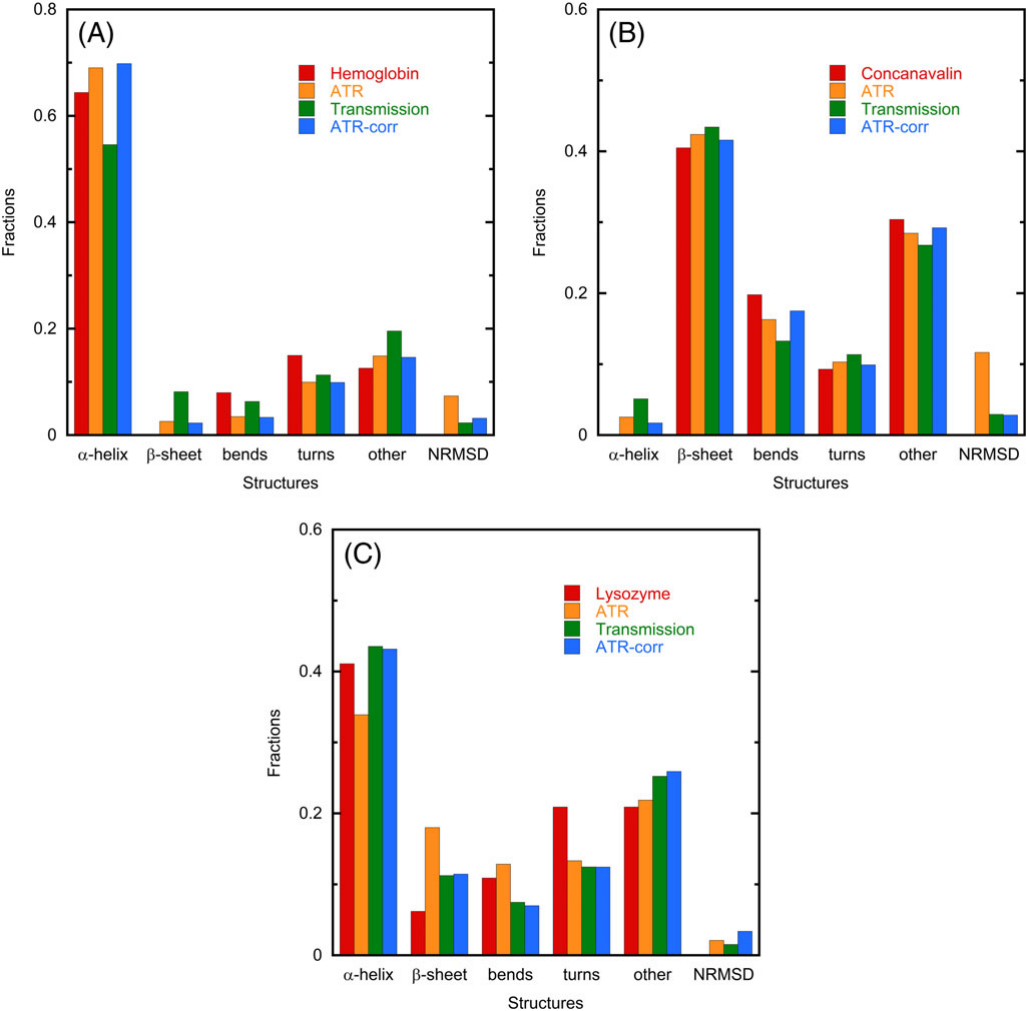
SOMSpec structure estimates for the data of Figures 2 and 3 as fractions of 1 compared with crystal structure estimates from the Protein Data Bank (missing bars denote 0%).^16^, ^17^, ^18^ The spectral normalized root mean squared deviation (NRMSD) is defined in the text. ATR, attenuated total reflectance

By way of contrast to the IR situation, CD secondary structure estimation by fitting to idealized spectra for different structural motifs is generally recognized to be a poor way of proceeding, and various different methods have been developed for comparing a sample spectrum with a reference set of spectra for proteins of known structure.^2^, ^3^, ^19^, ^20^, ^21^, ^22^ We recently developed a self‐organizing map neural network CD structure fitting methodology, SSNN,^4^ and validated it by leave‐one‐out comparisons with SELCON3^2^ and CDsstr^22^ using established reference sets that cover the structural space. We could see no conceptual or fundamental reason why such an approach should not be appropriate for IR absorbance data. The availability of a reference set covering the structural space is essential for such an approach. In contrast to CD, there are currently not many to choose from, so we used the BioTools Inc (Jupiter, Florida) reference set and an improved generalized version of our CD SSNN code, which we call SOMSpec, to extract secondary structure estimates from IR data.^23^ Figure 5 shows the output from SOMSpec for the data of Figures 1 and 2 alongside the Protein Data Bank crystal estimates, and the spectral normalized root mean squared deviation (NRMSD) defined as

(7) $$\text{NRMSD}=\sqrt{\left(\sum_{i}\left(x_{i,\text{experiment}}-x_{i,\text{model}}\right)/N\right)}/\left(M-m\right),$$

where *x*_*i*_ is the value at each wave number, *N* is the number of data points, *M* is the largest intensity, and *m* is the smallest, that gives a numerical measure of the goodness of spectral fit is also shown. The spectra NRMSD may or may not correlate to goodness of structural fit, but a large value calls the structural fit into question.

In Figure 5, the red bars correspond to the crystal structures, the orange to the uncorrected ATR data, the green to our transmission data, and blue to our corrected ATR spectra. The general conclusion to be made is that all three types of spectrum with SOMSpec are indicative of the secondary structures of the three exemplar proteins we have chosen. For the highly helical or sheet proteins, the ATR‐corr results are not significantly better than the original ATR data. For lysozyme, there is an improved performance although the NRMSD of the ATR‐corr is worse. Inspection of the output (data not shown) suggests that the NRMSD is more due to poor water vapor correction than structure. Figure 5A also supports our reservations about our own ability to buffer correct transmission spectra.

## 4 CONCLUSION

We have shown that ATR‐IR absorbance data of aqueous protein samples can be collected using a standard TGS detector and a ZnSe ATR unit. The data collection and baseline correction with ATR‐IR are much simpler than transmission spectroscopy in our hands. In ATR spectroscopy, the light intensity at the surface of the crystal depends on the refractive index, and hence absorbance, of the sample and in addition, the light intensity decays exponentially away from the surface in a manner that depends on the refractive index of the sample. We corrected for these effects by determining a wave number–dependent correction factor for the ATR spectra. To implement this simply, we approximated our Kramers‐Krönig–determined refractive index of water as a sum of four linear components across the amides I and II regions of the spectrum and used this for the proteins and water in which they were dissolved to determine the wave number–dependent correction factors. We simplified the correction factor by using an ATR angle of incidence of 45°. We then normalized the resulting spectra and applied our self‐organizing map secondary structure fitting algorithm, SOMSpec, to the amide I data using the BioTools reference set to give secondary structure estimates. The resulting secondary structure estimates are encouraging for the future of ATR spectroscopy for this purpose. In our hands, the results were at least as good as those for our transmission data. For highly α‐helical or β‐strand proteins, the uncorrected ATR spectra can be used. For mixed structures, based on our experience with lysozyme, the correction is necessary.

The data collection and new fitting methodology result in a new method for using an old technique to compare nominally the same biopharmaceutical products from different production methodologies, as well as giving secondary structure estimates for samples that for various reasons (high concentration of protein or buffer components) are intractable for CD measurements. As the chirality of proteins is well defined, the sensitivity of CD to the helical character of the proteins is not required, and the dependence of the amide I band on the backbone structure is sufficient for this purpose.

The sample in our ATR experiments has the area of the surface of the ATR crystal, which is approximately 1 mm^2^. If we can collect the data presented herein at 10 mg/mL with this area, then one can envisage in the future reducing the area and obtaining spatial resolution for protein IR experiments, which would enable molecular structural data to be extracted for different regions of a protein sample. Given the relative ease of the baseline correction with aqueous ATR compared with transmission IR absorption, we advocate ATR‐IR for such developments.

## Supporting information

Data S1. Supporting information
